# Powdered Sugar Examination as a Tool for the Assessment of *Paenibacillus larvae* Infection Levels in Honey Bee Colonies

**DOI:** 10.3389/fvets.2022.853707

**Published:** 2022-04-14

**Authors:** Stefano Bassi, Giorgio Galletti, Emanuele Carpana, Stefano Palminteri, Filippo Bosi, Giulio Loglio, Elena Carra

**Affiliations:** ^1^Istituto Zooprofilattico Sperimentale della Lombardia e dell' Emilia Romagna “Bruno Ubertini”, Brescia, Italy; ^2^Consiglio per la ricerca in agricoltura e l'analisi dell'economia agraria, Centro di ricerca Agricoltura e Ambiente, Bologna, Italy; ^3^Azienda Unità Sanitaria Locale di Bologna, Dipartimento di Sanità Pubblica, Bologna, Italy; ^4^Azienda Unità Sanitaria Locale della Romagna, Dipartimento di Sanità Pubblica, Ravenna, Italy; ^5^Agenzia di Tutela della Salute di Bergamo-Dipartimento di Prevenzione Veterinario, Bergamo, Italy

**Keywords:** *Paenibacillus larvae*, American Foulbrood, powdered sugar, assessment of infection levels, American Foulbrood control

## Abstract

American Foulbrood (AFB) is a contagious and severe brood disease of honey bees caused by the spore-forming bacterium *Paenibacillus larvae*. The identification of honey bee colonies infected by *P. larvae* is crucial for the effective control of AFB. We studied the possibility of identifying the infection levels by *P. larvae* in honey bee colonies through the examination of powdered sugar samples collected in the hives. The powdered sugar was dusted on the top bars of honeycombs and collected from a sheet paper placed at the bottom of the hive. Three groups of honey bee colonies were examined: *Group A1*- colonies with clinical symptoms of AFB (*n* = 11); *Group A2* – asymptomatic colonies located in apiaries with colonies showing symptoms of AFB (*n* = 59); *Group B* – asymptomatic colonies located in apiaries without cases of the disease (*n* = 49). The results showed that there was a significant difference in spore counting between *Groups* and that the spore load in sugar samples was always consistent with the clinical conditions of the colonies and with their belonging to AFB-affected apiaries or not. Based on the obtained results the cultural examination of powdered sugar samples collected from hives could be an effective tool for the quantitative non-destructive assessment of *P. larvae* infections in honey bee colonies.

## Introduction

American Foulbrood (AFB), which is caused by spore-forming bacterium *Paenibacillus larvae* (1), is the most widespread and severe bacterial disease affecting honey bee brood and causes considerable economic losses to beekeepers worldwide (2).

Clinically, the disease is characterized by darkened brood combs with a mottled appearance; a sour smell, greasy, sunken and perforated cell caps, but the pathognomonic symptom is the transformation of dead larvae into a ropy mass that forms a characteristic viscous thread when a matchstick is inserted into the cell and then pulled out.

If not removed, the dead larvae dries into a blackish scale tightly adhering to the wall of the cells (3).

The diagnosis of AFB is based on the recognition of typical symptoms and on the identification of *P. larvae* by means of laboratory techniques (4).

The molecular characterization of *P. larvae* strains via repetitive element PCR (rep-PCR) using enterobacterial repetitive intergenic consensus (ERIC) primers allows for the identification of five different ERIC genotypes designated as ERIC I-V (1, 5).

Genotype ERIC V was isolated only once from a Spanish honey sample (5), while the genotypes ERIC III and ERIC IV have been isolated very rarely and in the past and have little practical importance (6). The genotypes ERIC I and ERIC II instead are those regularly isolated from AFB outbreaks in Europe and worldwide (1, 7–11). These two genotypes have different phenotypic characteristics and differ, above all, in the morphology/pigmentation of colonies and in virulence (1).

The colonies of *P. larvae* genotype ERIC II are characterized by a typical orange pigmentation that is never observed in the colonies of genotype ERIC I (1, 10, 12).

Regarding virulence, strains of *P. larvae* genotype ERIC II are more virulent at the larval level than strains of genotype ERIC I, and all infected larvae are killed within 6-7 days instead of within 13–15 days. Therefore, most larvae die before cell capping, and adult bees can easily remove dead larvae from their hives. There are few larvae that die after cell capping; consequently, the darkened and perforated caps can be very low in number. Identifying decaying larvae is more difficult than in the case of ERIC I genotype infections, and the risk of overlooking the disease, especially in the early stages, is considerable (13, 14).

Križanová et al. (15) and Drobniková et al. (16) described outbreaks of AFB with atypical symptoms caused by orange-pigmented *Bacillus larvae* strains in the Czech and Slovak Republics. In these outbreaks, the following atypical symptoms were observed: (*a*) the caps of diseased brood cells were not darkly colored; (b) decaying larvae were light brown or gray in color without a glue-like consistency but instead with a watery consistency; (c) light brown or gray scales were easily removed from the cells; (d) white larvae protruding from uncapped cells could be seen; (e) sometimes the larvae had an appearance similar to what is observed in Sacbrood disease.

Outbreaks of AFB caused by *P. larvae* genotype ERIC II with the same or very similar symptoms are sometimes observed also today in northern Italy (personal observation).

When the darkened and perforated caps are very low in number or atypical symptoms are present the clinical diagnosis of AFB is, of course, more difficult.

American Foulbrood generally occurs when a certain level of contamination by *P. larvae* spores in honey bee colonies is reached (17); nevertheless, some colonies can contain even a relatively large number of spores over several seasons without showing clinical symptoms (18–21).

These colonies contribute, first of all, to the transmission of *P. larvae* spores from one hive to another (22); furthermore, they can more or less quickly develop the disease.

The identification of infected but asymptomatic colonies can be performed through a search for *P. larvae* spores in materials taken from hives, and standard methods based on microbiological and biomolecular techniques are available for the detection and quantification of the spore load in adult bees, honey from super, honey from the brood chamber, wax and wax debris (4, 23, 24).

The culture-based techniques for the detection of *P. larvae* in materials taken from the hive (honey, bees and debris) are a good alternative to the PCR methods. The results obtained with the cultural methods are often more accurate than those obtained with PCR. This may depend on a variety of factors, such as the presence of polymerase inhibitors in some hive materials and/or failures in the DNA extraction from *P. larvae* spores (25).

We studied the possibility of identifying honey bee colonies infected by *P. larvae* at various levels using the powdered sugar dusting technique.

The powdered sugar is frequently used in beekeeping practices for various purposes.

The powdered sugar roll method performed on samples of adult bees is used to monitor Varroa infestation levels (26, 27). This approach can be used to determine further treatment needs or to assess past treatment effectiveness.

The dusting of bees with powdered sugar, removing mites from adult bees, has been used also for the control of Varroa infestations (28, 29).

Another use of this material occurs in the therapy of some diseases where the drugs are mixed with powdered sugar before the dusting on the tops of the combs.

Icing sugar is not toxic to adult bees and bee brood (30) moreover is practice to use and inexpensive.

In this work we have dusted the powdered sugar on the top bars of the combs and the sugar collected at the hive bottom, was examined by a culture method for *P. larvae* spore counting.

The results of cultural examinations were compared to the outcomes of clinical inspections performed at the time of sugar sampling to study relationships between the number of spores in the sugar samples and the clinical status of the colonies or apiaries.

## Materials and Methods

### Apiaries and Clinical Inspection

The work was carried out on 119 colonies belonging to 10 apiaries located in the Emilia-Romagna and Lombardy regions of northern Italy. The number of hives ranged from 4 to 17 per apiary (Tables 1, 2).

**Table 1:** Apiaries (***Groups A1 and A2***) with recurrent cases of overt AFB reported in recent years: results of clinical inspections, cultural examinations and genotype of *P. larvae* isolates.

**Apiary**	**N. Colonies**	Group of colonies on the basis of AFB symptoms ^*^	**N. of colonies with different classes of contamination (CFU/g)**						**Genotype isolated**
			**Class 1 (< LOD)**	**Class 2 (|-100)**	**Class 3 (|-1,000)**	**Class 4 (|-10,000)**	**Class 5 (|-100,000)**	**Class 6 (≧100,000)**	
1	15	**A1 (1)**						1 (304,000)	ERIC I
		**A2 (14)**	5 (<20)	4 (20) 1 (40)	1 (320) 1 (400)	1 (2,600) 1 (7,700)			
2	14	**A1 (1)**						1 (200,000)	ERIC I
		**A2 (13)**	8 (<20)	3 (20) 2 (40)					
3	17	**A1 (1)**						1 (4,440,000)	ERIC II
		**A2 (16)**	3 (<20)	1 (20) 1 (40) 1 (60) 2 (80)	1 (100) 1 (200) 1 (220) 1 (460) 1 (560)	1 (2,700) 1 (2,420)	1 (17,980)		
4	17	**A1 (5)**					1 (44,000) 1 (52,800) 1 (54,200)	1 (128,000) 1 (3,000,000)	ERIC II
		**A2 (12)**	1 (<20)	1 (20) 1 (40) 1 (60) 1 (80)	1 (200) 1 (240) 1 (260) 1 (560) 1 (680) 1 (700)	1 (3,680)			
5	7	**A1 (3)**						1 (256,000) 1 (380,000) 1 (5,000,000)	ERIC I
		**A2 (4)**		1 (60)		1 (6,220)	1 (17,000) 1 (21,400)		

* ***Group A1**, Colonies with clinical symptoms of AFB. **Group A2**, Colonies without clinical symptoms of AFB*.

**Table 2:** Apiaries (***Group B***) without cases of AFB reported in recent years: results of clinical inspections, cultural examinations and genotype of *P. larvae* isolates.

**Apiary**	**N. Colonies**	**N. Colonies with AFB symptoms**	**N. of colonies with different classes of contamination (CFU/g)**						**Genotype isolated**
			**Class 1 (< LOD)**	**Class 2 (|-100)**	**Class 3 (|-1,000)**	**Class 4 (|-10,000)**	**Class 5 (|-100,000)**	**Class 6 (≧100,000)**	
6	10	0	7 (<20)	1 (20) 1 (40)	1 (120)				ERIC II
7	15	0	11 (<20)	1 (20) 1 (40) 1 (80)	1 (140)				ERIC I
8	10	0	9 (<20)	1 (80)					ERIC I
9	10	0	10 (<20)						
10	4	0	4 (<20)						

The apiaries were characterized by different histories in terms of the presence of AFB. In five apiaries (*Groups* A1 and A2), cases of recurring AFB were reported in years previous to this work, whereas in the other five apiaries (*Group* B), which were tested as a control group, no cases of AFB had been reported in the last years.

Before sampling, each colony was checked via clinical examination. Visual inspection was carried out on all brood combs, and the suspected cells were inspected to detect ropy larval remains or scales. Additionally, dead larvae in capped or uncapped cells with atypical lesions (e.g., a watery consistency) were examined. The clinical diagnosis was confirmed after the laboratory isolation of *P. larvae* from larval remains or scales. The results of clinical examinations were recorded as denoting the presence or absence of disease symptoms.

### Sampling Procedures

In each hive, 50 g of powdered sugar containing 3% corn starch was dusted on the top bars of the combs in the brood chamber and immediately brushed between the frames. After 20–25 min, the sugar was collected from a sheet paper previously placed on the hive bottom and transferred into a plastic jar with a screw cap. The jars were sent to the laboratory and stored at 3°C until analysis.

After powdered sugar sampling, all the diseased colonies were killed and burned in accordance with national law.

### Microbiological Analysis

In brief, 1 g of sugar was placed in a 15 ml test tube with a screw cap containing 9 ml of sterile distilled water. The test tube was shaken by hand until the sugar dissolved and then heated in a water bath at 85°C for 15 min to inactivate the thermosensitive contaminants. The solution was plated on five Petri dishes (100 μl/plate) of MYPGP (Mueller-Hinton broth, yeast extract, potassium phosphate, glucose and pyruvate) agar supplemented with nalidixic and pipemidic acid.

The plates were incubated at 37 °C in an atmosphere with 10% CO_2_ and examined after 3 and 8 days.

When counting was not possible due to the presence of very large numbers of colonies or to the rapid growth of spore-forming bacteria, ten-fold dilutions from the initial solution were prepared and then cultured with the previously described procedures.

For each sample, one to five colonies with *P. larvae*-like morphology were tested for catalase reactions and subjected to Gram staining, always resulting in catalase negativity with Gram-positive rods.

One or two strains for each sample were cultured on TSYEA (tryptic soy yeast extract agar) slant and stored at 2 °C for further molecular analysis.

After the numeration of *P. larvae* colonies, the number of viable spores was calculated and expressed as colony forming units (CFU). The limit of detection (LOD) of the method was 20 CFU/g.

### Molecular Analysis

The strains stored on TSYEA slant were tested by PCR according to Dobbelaere et al. (31), and all strains were identified as *P. larvae*.

In the sugar samples positive for *P. larvae*, the colony morphology was always uniform, and only one isolate per sample was genotyped by ERIC-PCR using primers applied by [Genersch and Otten (32)] and following the PCR protocol described by Bassi et al. (10).

### Data Analysis

Descriptive statistics (absolute frequencies and percentages) were obtained regarding the number of colonies with different contamination levels. Data “ < LOD” were treated as 10 CFU/g for calculation purposes. The Kruskal-Wallis test was used to compare spore counts in *Groups* identified with clinical inspection. R 4.0.2 (33) and Microsoft Excel for Windows were used to manage the data.

## Results

The results of clinical inspections, spore counting carried out on the powdered sugar samples, and ERIC genotyping are reported in Tables 1, 2.

The clinical controls in the five apiaries with recurrent AFB outbreaks highlight 11 colonies with AFB symptoms and 59 asymptomatic colonies (Table 1).

The clinical controls in five apiaries with no cases of AFB in recent years showed no symptoms of AFB (Table 2).

The diagnostic controls allowed us to divide the 119 colonies into 3 groups: *Group A1*-colonies with clinical symptoms of AFB (*n* = 11); *Group A2*-colonies without clinical symptoms located in apiaries with colonies showing symptoms of AFB (*n* = 59); and *Group B*-colonies without symptoms of AFB and located in apiaries where no cases of the disease were reported in the last years (*n* = 49).

In the AFB-diseased apiaries, the spore load in symptomatic colonies (*Group A1*) ranged from 4.4 x 10^4^ to 5.0 x 10^6^ CFU/g with a mean value of 1,259,909 (sd = 1,913,607). In the non-symptomatic colonies (*Group A2*), the presence of *P. larvae* spores was not demonstrated in 17 samples, and in the remaining 42 samples (71.2, 95% CI 57.9 – 82.2), the spore load ranged from 2.0 x 10^1^ to 2.1 x 10^4^ CFU/g with an average number of spores per sugar sample of 2,081 CFU/g (sd = 4,993) (Table 1).

In the apiaries of *Group B*, the presence of *P. larvae* spores was not observed in 41 sugar samples; in the remaining 8 samples (16.3, 95% CI 7.3–29.7), the spore load ranged from 2.0 x 10^1^ to 1.4 x1 0^2^ CFU/g, and the mean number of spores per sugar sample reached 67.5 CFU/g (sd=45.3) (Table 2).

**Figure 1:**
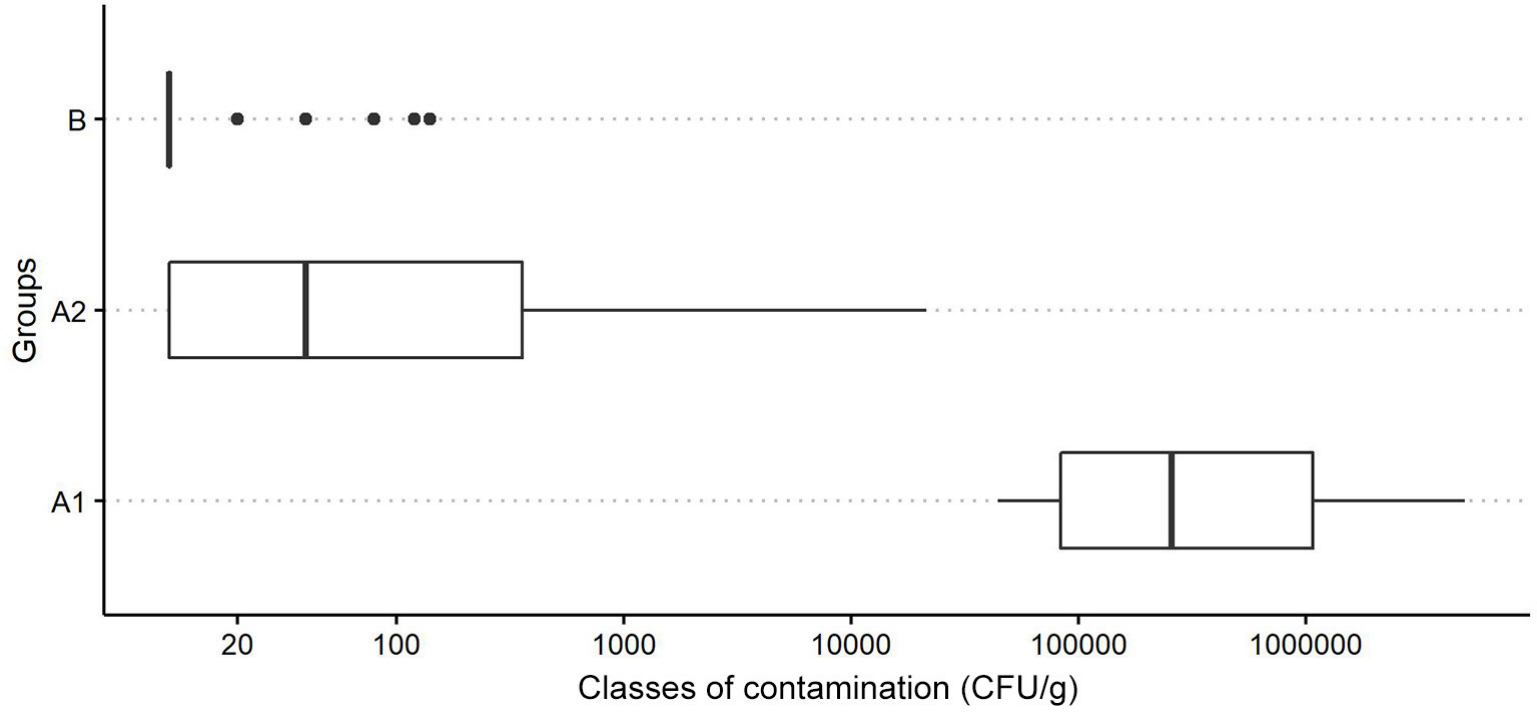
Boxplot of the distribution of *P. larvae* spore contamination classes (CFU/g) in powdered sugar samples for different groups (A1, colonies with clinical symptoms of AFB; A2, colonies without clinical symptoms of AFB located in apiaries with colonies showing symptoms of AFB; and B: colonies from apiaries with no cases of AFB in the last years-control group).

The distribution of *P. larvae* spore contamination levels (CFU/g) in these *Groups* is reported in Figure 1. The Kruskal-Wallis test shows a significant difference in spore counts between the *Groups* (H = 59.2, *p* < 0.01).

**Figure 2:**
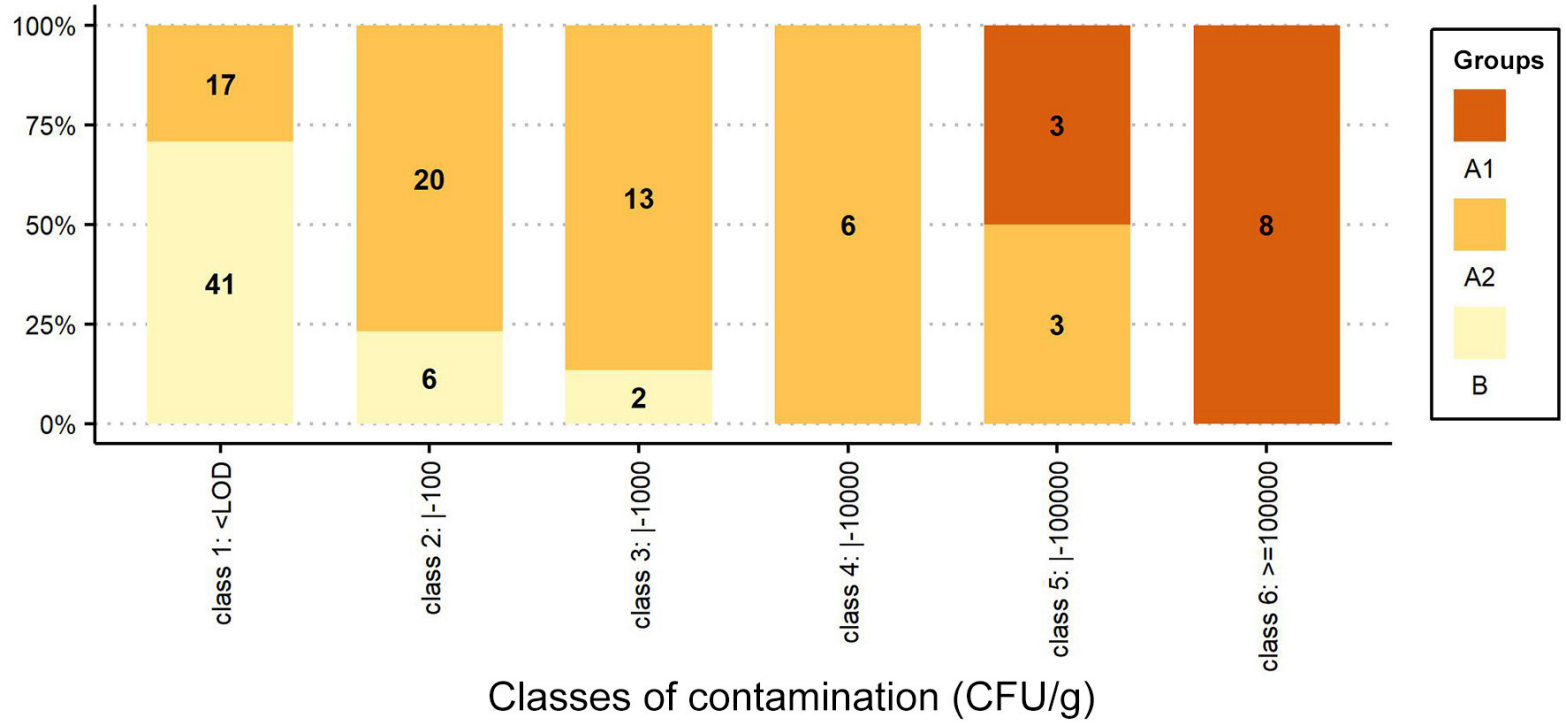
Percentage distribution of samples/colonies grouped by classes of contamination (CFU/g). The number of samples/colonies is shown inside each bar.

In Figure 2, the percentage distributions of samples/colonies are grouped into six classes of contamination (class 1: < LOD; class 2: <100; class 3: <1,000; class 4: <10,000; class 5: <100,000, and class 6: ≤ 1,000,000 CFU/g), and the number of samples/colonies belonging to each *Group* is reported inside each bar.

The distribution of the results in terms of contamination classes (Figure 2) highlights the differences, reported in Figure 1, between the *Groups*: all symptomatic colonies of the AFB-diseased apiaries (*Group A1*) fall under classes 5 and 6 (3 and 8 colonies, respectively); the asymptomatic colonies (*Group A2*) span different size classes (for classes 1 to 5, we find 17, 20, 13, 6 and 3 colonies, respectively); the asymptomatic colonies belonging to the control group (*Group B*) are mainly of the first class (41 colonies), and the 8 positive samples are of classes 2 and 3 (6 and 2 colonies, respectively).

The ERIC genotyping of the selected strains shows that in the AFB-diseased apiaries, *P. larvae* genotype ERIC I was isolated in three apiaries (1–2–5), and *P. larvae* genotype ERIC II was isolated in two apiaries (3–4). No mixed infections were observed in the same hive or apiary (Table 1).

In the apiaries without cases of AFB, genotype ERIC I was isolated from the powdered sugar in two apiaries (7–8), and genotype ERIC II was isolated from one apiary (6). In the remaining two (9–10), *P. larvae* were not isolated (Table 2).

## Discussion and Conclusion

The aim of this work was to study the performance of powdered sugar samples collected from hives in detecting *P. larvae* spore load in honey bee colonies.

Powdered sugar samples were taken from apiaries with different clinical conditions and history.

The results show that the *P. larvae* spore numbers in the sugar samples were clearly related to the presence or absence of the disease in the colonies or apiaries.

As highlighted by our boxplot graph (Figure 1), the difference in the spore loads of the AFB-diseased apiaries and non-diseased apiaries is notable.

In the AFB-diseased apiaries, the number of *P. larvae* spores in sugar samples collected from colonies with overt AFB *(Group A1)* was always greater than for the asymptomatic colonies *(Group A2)*, although some (3/42) asymptomatic colonies showed spore numbers very close to those of symptomatic colonies (Table 1) and fell under size class 5 together with diseased colonies (Figure 2).

The spore loads observed in the symptomatic colonies *(Group A1)* were significantly higher than those found in the colonies of the control group *(Group B)*, and the results always fall under different size classes (Figure 2).

Taking into consideration only colonies without symptoms, the comparison between the *Group* A2 (apiaries including diseased colonies) and the *Group* B (healthy apiaries) shows a significant higher spores level in colonies of the *Group* A2, in addition to a much higher proportion of positive values (71 vs. 14%). This can be explained by the fact that in the apiaries with AFB cases the spread of *P. larvae* spores to healthy colonies inevitably takes places, as common beekeeping practices facilitate horizontal transfer of pathogens, especially if numerous hives are managed in close proximity (34).

The bacterial spore load in the sugar samples is always consistent with the clinical conditions of colonies and with whether they belong to AFB diseased apiaries or not.

These results show that the cultural examination of powdered sugar samples collected from hives allows us to detect infection levels by *P. larvae* spores of colonies.

Knowledge of infection levels allows for the adoption of appropriate management strategies to prevent both AFB onset and the transmission of infection between colonies and apiaries (35).

The identification of colonies with high *P. larvae* spore levels can be useful not only for preventive purposes but also for diagnostic purposes.

The clinical diagnosis of AFB is typically based on the recognition of symptoms by visual inspection. However, field inspection, particularly for large beekeeping operations, is laborious and time consuming; moreover, the obtained results are highly operator dependent (36).

It should be added that by visual inspection in early stages, the disease may remain undetectable (22), and in any case, clinical examination does not enable the detection of colonies with asymptomatic infections.

The identification of colonies with significant spore loads used as a screening/diagnostic tool allows for clinical checks only of suspected colonies, reducing field labor requirements.

Moreover, targeted checks improve clinical examination performance, facilitating the identification of colonies with very few suspected cells or with atypical lesions that can escape a “routine” clinical examination.

Powdered sugar has some advantages over other materials used to monitor *P. larvae* infections in honey bee colonies. The sampling is not destructive and is very easy to do, the packaging, the transportation to the laboratory and the sample handling in laboratory are very practical to perform.

Furthermore, the powdered sugar examination, as well as the examination of adult bees (25), provides a “snapshot” of the actual infection status unlike that occurs by examining other materials, such as the debris collected during the wintering period or the honey, that reflect instead the historical accumulation of bacterial spores over time.

Based on the results of our trial, the cultural examination of powdered sugar samples collected from hives seems to be an effective tool for the quantitative non-destructive assessment of *P. larvae* infections in honey bee colonies.

## Data Availability Statement

The original contributions presented in the study are included in the article/supplementary material, further inquiries can be directed to the corresponding author/s.

## Author Contributions

SB: conceptualization, methodology, investigation, data collection and analysis, writing the original draft, review and editing, and supervision. GL, FB, and SP: investigation and supervision. ECarp: conceptualization, review and editing the original draft, and supervision. GG: data analysis and review and editing the original draft. ECarr: investigation, data collection and analysis, review and editing the original draft, and supervision. All authors contributed to the article and approved the submitted version.

## Conflict of Interest

The authors declare that the research was conducted in the absence of any commercial or financial relationships that could be construed as a potential conflict of interest.

## Publisher's Note

All claims expressed in this article are solely those of the authors and do not necessarily represent those of their affiliated organizations, or those of the publisher, the editors and the reviewers. Any product that may be evaluated in this article, or claim that may be made by its manufacturer, is not guaranteed or endorsed by the publisher.

## References

[1] Genersch E Forsgren E Pentikäinen J Ashiralieva A Rauch S Kilwinski J. Reclassification of Paenibacillus larvae subsp. pulvifaciens and Paenibacillus larvae subsp larvae as Paenibacillus larvae without subspecies classification. Int J Syst Evol Microbiol. (2006) 56:501–11. 10.1099/ijs.0.63928-0 16514018

[2] Ellis JD Munn PA. The worldwide health status of honey bees. Bee World. (2005) 86:88–101. 10.1080/0005772X.2005.11417323

[3] Hansen H Brødsgaard CJ. American foulbrood: a review of its biology, diagnosis and control. Bee World. (1999) 80:5–23. 10.1080/0005772X.1999.1109941524761740

[4] Anonymous. American foulbrood of honey bees (Infection of honey bees with *Paenibacillus larvae*). In: OIE Manual of Diagnostic Tests and Vaccines for Terrestrial Animals, Chapter 3.2.2. (2018). p. 719–35. Available online at: https://www.oie.int/fileadmin/Home/eng/Health_standards/tahm/3.02.02_AMERICAN_FOULBROOD.pdf.

[5] Beims H Bunkc B Erlerd S Mohre K Spröerc C Pradellac S. Discovery of *Paenibacillus larvae* ERIC V: Phenotypic and genomic comparison to genotypes ERIC I-IV reveal different inventories of virulence factors which correlate with epidemiological prevalences of American Foulbrood. Int J Med Microbiol. (2020) 310:151394. 10.1016/j.ijmm.2020.151394 31959580

[6] Genersch E. American foulbrood in honey bees and its causative agent, *Paenibacillus larvae*. J Invert Pathol. (2010) 103:10–9. 10.1016/j.jip.2009.06.015 19909971

[7] Pentikäinen J Kalliainen E Pelkonen S. Molecular epidemiology of *Paenibacillus larvae* infection in Finland. Apidologie. (2009) 40:73–81. 10.1051/apido:2008061

[8] Loncaric I Derakhshifar I Oberlerchner JT Köglberger H Moosbeckhofer R. Genetic diversity among isolates of *Paenibacillus larvae* from Austria. J Invertebr Pathol. (2009) 100:44–6. 10.1016/j.jip.2008.09.003 18831978

[9] Schäfer MO Genersch E Fünfhaus A Poppinga L Formella N Bettin B. Rapid identification of differentially virulent genotypes of *Paenibacillus larvae*, the causative organism of American foulbrood of honey bees, by whole cell MALDI-TOF mass spectrometry. Vet Microbiol. (2014) 170:291–7. 10.1016/j.vetmic.2014.02.006 24613082

[10] Bassi S Formato G Milito M Trevisiol K Salogni C Carra E. Phenotipic characterization and ERIC-PCR based genotyping of *Paenibacillus larvae* isolates recovered from American foulbrood outbreaks in honey bees from Italy. Vet Q. (2015) 35:27–32. 10.1080/01652176.2014.993095 25431956

[11] Biová G Bzdil J Dostálková S Pet?rivalský N Brus J Carra E. American foulbrood in the Czech Republic: ERIC II genotype of *Paenibacillus larvae* is prevalent. Front Vet Sci. (2021) 8:698976. 10.3389/fvets.2021.698976 34485429 PMC8416417

[12] Hirai Y Suzuki T Inaba N Minoguchi N Takamatsu D. Existence of *Paenibacillus larvae* genotypes ERIC I-ST2, ERIC I-ST15 and ERIC II-ST10 in the western region of Aichi prefecture, Japan. J Vet Med Sci. (2016) 78:1195–9. 10.1292/jvms.16-0041 27020320 PMC4976278

[13] Genersch E. *Paenibacillus larvae* and American Foulbrood long since known and still surprising. J Verbr Lebensm. (2008) 3:429–34. 10.1007/s00003-008-0379-8

[14] Genersch E. Amerikanische Faulbrut: oft anders als im Lehrbuch. Deutsches Bienen J. (2009) 8:4–6.

[15] KriŽanová H Halaša N Roško L Zubaj J. Results of studies on the causative agent of American foulbrood. Vet Med. (1988) 33:633–40.

[16] Drobniková V Richter V Häusler J Pytelová I. Characterization of *Bacillus larvae* and related bacilli by chromatography of cell fatty acids. J Apic Res. (1994) 33:69–74. 10.1080/00218839.1994.11100852

[17] Anonymous. Report of the Meeting of the OIE ad hoc Group on Diseases of Honey Bees.- January 31 - February 2, Paris, France. The World Organisation for Animal Health, OIE (2012). Available online at: https://www.oie.int/fileadmin/Home/eng/Internationa_Standard_Setting/docs/pdf/SCAD/A_SCAD_Feb2012.pdf.

[18] Hansen H Rasmussen B. The investigation of honey from bee colonies for *Bacillus larvae*. Dan J Plant Soil Sci. (1986) 90:81–6.

[19] Hornitzky MAZ Clark S. Culture of *Bacillus larvae* from bulk honey samples for the detection of American foulbrood. J Apic Res. (1991) 30:13–6. 10.1080/00218839.1991.11101228

[20] Steinkraus KH Morse RA. American foulbrood incidence in some US and Canadian honeys. Apidologie. (1992) 23:497–501. 10.1051/apido:19920601

[21] Fries I Lindström A Korpela S. Vertical transmission of American foulbrood (*Paenibacillus larvae*) in honey bees (Apis mellifera). Vet Microbiol. (2006) 114:269–74. 10.1016/j.vetmic.2005.11.068 16420974

[22] Lindström A Fries I. Sampling of adult bees for detection of American foulbrood (*Paenibacillus larvae* subsp *larvae)* spores in honey bee (Apis mellifera) colonies. J Apic Res. (2005) 44:82–6. 10.1080/00218839.2005.11101154

[23] Von der Ohe W Dustmann JH. Efficient prophylactic measures against 8 American foulbrood by bacteriological analysis of honey for spore contamination. Am Bee J. (1997) 137:603–6.

[24] de Graaf DC Alippi AM Antúnez K Aronstein KA Budge G De Koker D. Standard methods for American foulbrood research. In: Dietemann V, Ellis JD, Neumann P, Editors. The Coloss Beebook, Volume II: Standard Methods For Apis Mellifera Pest And Pathogen Research. (2013). p. 52. 10.3896/IBRA.1.52.4.16

[25] Forsgren E Laugen AT. Prognostic value of using bee and hive debris samples for the detection of American foulbrood disease in honey bee colonies. Apidologie. (2014) 45:10–20. 10.1007/s13592-013-0225-6

[26] Macedo PA Wu J Ellis Marion D. Using inert dusts to detect and assess varroa infestations in honey bee colonies. J Apic Res. (2002) 40:3–7. 10.1080/00218839.2002.11101062

[27] Gregorc A Knight PR Adamczyk J. Powdered sugar shake to monitor and oxalic acid treatments to control varroa mites (Varroa destructor Anderson and Trueman) in honey bee (Apis mellifera) colonies. J Apic Res. (2017) 56:71–5. 10.1080/00218839.2017.1278912

[28] Aliano NP Ellis MD. A strategy for using powdered sugar to reduce varroa populations in honey bee colonies. J Apic Res. (2005) 44:54–7. 10.1080/00218839.2005.11101148

[29] Stanimirovic Z Aleksi N Stevanovi J Cirkovic D Mirilovic Djelic N. The influence of pulverised sugar dusting on the degree of infestation of honey bee colonies with Varroa destructor. Acta Veterinaria. (2011) 61:309–25. 10.2298/AVB1103309S

[30] Pettis JS Kochansky J Feldlaufer MF. Larval *Apis mellifera* L. (Hymenoptera: Apidae) mortality after topical application of antibiotics and dusts. J Econ Entomol. (2004) 97:171–6. 10.1603/0022-0493-97.2.171 15154433

[31] Dobbelaere W de Graaf DC Peeters JE Jacobs FJ. Development of a fast and reliable diagnostic method for American foulbrood disease (*Paenibacillus larvae* subsp. larvae) using a 16S rRNA gene based PCR. Apidologie. (2001) 32:363–70. 10.1051/apido:2001136

[32] Genersch E Otten C. The use of repetitive element PCR fingerprinting (rep-PCR) for genetic subtyping of German field isolates of Paenibacillus larvae subsp. larvae Apidologie. (2003) 34:195–206. 10.1051/apido:2003025

[33] Core Team R. R: A language environment for statistical computing. Vienna: R Foundation for Statistical Computing (2018). Available online at: URL https://www.R-project.org/.

[34] Fries I Camazine S. Implications of horizontal and vertical pathogen transmission for honey bee epidemiology. Apidologie. (2001) 32:199–214. 10.1051/apido:200112225079600

[35] Locke B Low M Forsgren E. An integrated management strategy to prevent outbreaks and eliminate infection pressure of American foulbrood disease in a commercial beekeeping operation. PrevVet Med. (2019) 167:48–52. 10.1016/j.prevetmed.2019.03.023 31027721

[36] Lyall D Hansbro P Horvat J Stanwell P. Quantitative nondestructive assessment of paenibacillus larvae in apis mellifera hives. In: Ahram T, Karwowski W, Pickl S, Taiar R, editors. Human Systems Engineering and Design II: Proceedings of the 2nd International Conference on Human Systems Engineering and Design (IHSED2019): Future Trends and Applications. Munich: Springer International Publishing (2019). p. 579–83. 10.1007/978-3-030-27928-8_88

